# Quality of Life as a Predictor of Successful Aging in Urban and Rural Older Adults: A Cross-Sectional Study in Eastern Croatia–Slavonia

**DOI:** 10.3390/healthcare14030296

**Published:** 2026-01-24

**Authors:** Marija Barišić, Ivana Barać, Jasenka Vujanić, Nikolina Farčić, Štefica Mikšić, Maja Čebohin, Robert Lovrić, Dunja Degmečić, Marko Krnjajić, Željka Dujmić, Željko Mudri

**Affiliations:** 1 Department of Nursing and Palliative Medicine, Faculty of Dental Medicine and Health Osijek, Josip Juraj Strossmayer University of Osijek, Car Hadrijan Street 21, 31000 Osijek, Croatia; mbarisic@fdmz.hr (M.B.); jvujanic@fdmz.hr (J.V.); nfarcic@fdmz.hr (N.F.); smiksic@fdmz.hr (Š.M.); mcebohin@fdmz.hr (M.Č.); m.krnjajic02@gmail.com (M.K.); zeljka.dujmic@bolnicasb.hr (Ž.D.); 2 Medical Faculty of Osijek, Josip Huttler Street 4, 31000 Osijek, Croatia; dunja.degmecic@mefos.hr; 3 University Hospital Centre Osijek, Josip Huttler Street 4, 31000 Osijek, Croatia; 4 Nursing Institute “Professor Radivoje Radić”, Faculty of Dental Medicine and Health Osijek, Josip Juraj Strossmayer University of Osijek, 31000 Osijek, Croatia; rlovric@fdmz.hr; 5 General Hospital “Dr. Josip Benčević” Slavonski Brod, Andrija Štampar Street 42, 35000 Slavonski Brod, Croatia; 6 Department of Sociology, Croatian Catholic University Zagreb, Ilica Street 242, 10000 Zagreb, Croatia

**Keywords:** quality of life, successful aging, older adults, urban and rural settings, cross-sectional study, Slavonia, Eastern Croatia

## Abstract

**Highlights:**

**What are the main findings?**

- Older adults in rural areas reported lower self-rated successful aging (SSAS) and personal wellbeing (PWI) than those in urban/suburban areas. PWI was the strongest positive predictor while rural residence and chronic illness were negative predictors.
- Active community involvement was positively associated with quality of life, whereas regret over missed opportunities or past actions had negative associations; no significant gender differences were observed in SSAS or PWI.

**What are the implications of the main findings?**

- Promoting social engagement and psychosocial support could improve successful aging in rural areas
- Considering wellbeing alongside health status and addressing rural disparities could improve aging programs.

**Abstract:**

**Background:** Population aging has increased attention on the quality of life and successful aging of older adults. **Objective:** To examine urban–rural differences in subjective quality of life and self-rated successful aging, explore associations with psychosocial factors, and identify predictors of successful aging, including potential moderating effects of place of residence and chronic illness. **Methods:** A cross-sectional study was conducted among 403 adults aged ≥ 60 years in Eastern Croatia. Measures included a sociodemographic questionnaire, the Self-assessment of Successful Aging Scale (SSAS), and the Personal Wellbeing Index (PWI). Data were analyzed using nonparametric tests (Mann–Whitney U, Spearman’s correlation), linear regression, and moderation analyses. Significance was set at *p* < 0.05. Ethical approval was obtained (Class: 602-01/24-12/02; IRB: 2158/97-97-10-24-36). **Results:** Rural participants reported lower PWI scores (*p* = 0.005) and self-rated successful aging (*p* < 0.001) than urban participants. Active community involvement was positively associated with quality of life (Rho = 0.46; *p* < 0.001), whereas regret about missed opportunities and past actions was negatively associated (Rho = −0.20; *p* < 0.01). Regression analyses explained 48.3% of the variance in SSAS, with higher PWI scores being strongly associated with higher SSAS scores, and rural residence and chronic illness being associated with lower SSAS scores. Moderation analyses indicated that the association between PWI and SSAS was consistent across different environmental contexts and in the presence of illness. **Conclusions:** Older adults living in rural areas reported lower quality of life and self-rated successful aging compared with those in urban and suburban areas, with subjective wellbeing emerging as a key predictor. Promoting social engagement and addressing psychosocial barriers may enhance successful aging, particularly in rural populations. Findings suggest that social engagement and psychosocial support are associated with higher level of perceived successful aging, indicating potential areas for future community-based or healthcare interventions.

## 1. Introduction

The pace of global population aging has accelerated markedly compared with previous decades. In 2020, the number of individuals aged 60 years and older surpassed the number of children under five [1], and current projections indicate that this demographic will increase from approximately one billion in 2020 to more than two billion by 2050, with one in six people worldwide expected to be aged 60 years or older by 2030 [2]. Concurrently, the population of adults aged 80 years and above is projected to triple, rising from about 140 million in 2020 to approximately 426 million by 2050 [1].

Although increased life expectancy is widely recognized as an indicator of biomedical, social, and economic advancement [3], population aging simultaneously poses a substantial challenge to the sustainability of these systems [4]. Accelerating demographic trends, coupled with systemic pressures, may compromise the living conditions and overall wellbeing of older adults. Population aging is therefore characterized not only by changes in the age structure of the population but also by shifts in the health and social care needs of older adults, the availability and organization of care systems, and the social dynamics shaped by the specific contexts in which older adults live.

Older adults are considered a high-risk group in terms of health and quality of life [5], being particularly vulnerable to chronic diseases, geriatric syndromes, and multimorbidity, which in turn increases the demand for healthcare services [6,7,8,9]. Moreover, they face a higher likelihood of requiring both formal and informal long-term care [10,11]. Therefore, it is crucial to ensure environments in which additional years of life are spent living in good health and within supportive settings, where individuals’ ability to remain functional, independent, and active is minimally constrained [12]. In this context, ensuring a high quality of life in later life occupies a central role.

### 1.1. Biopsychosocial Perspectives on Quality of Life in Older Adults

Quality of life is defined as an individual’s perception of their position in life in the context of the culture and value systems in which they live and in relation to their goals, expectations, standards, and concerns [13]. It is a complex, multidimensional construct encompassing physical health, psychological state, level of independence, social relationships, personal beliefs, and one’s relationship to key features of the environment. Quality-of-life assessment is inherently subjective and shaped by the context in which a person lives [13], reflecting the interplay of biological, psychological, and social factors [14]. Accordingly, the present study examines the subjective dimension of quality of life, with particular attention given to the personal wellbeing of older adults.

Although quality of life tends to decline in very old age, its association with chronological age is not linear, as periods of relative stability and even potential improvement may occur [15]. The process is highly dynamic and individualized [16], supporting an understanding of aging as a heterogeneous phenomenon characterized by concurrent losses and compensatory adaptations [17].

Given this dynamic and heterogeneous nature of aging, quality of life in older adults is examined within a biopsychosocial framework [5], extending beyond earlier approaches that focused solely on health status. Notably, self-perceived quality of life can remain high despite physical decline [14], underscoring its subjective and complex nature. Due to its multidimensionality, a variety of factors influence quality of life among older adults [16]. This framework conceptualizes quality of life as a dynamic process, in which daily opportunities and available resources shape individuals’ perceptions of their own aging. Subjective assessments are thus oriented toward incorporating factors and resources that enable older adults to perceive themselves as successful in the aging process, even amidst health or social limitations.

### 1.2. Successful Aging

Successful aging involves the preservation of physical, cognitive, and social functioning, along with the maintenance of high quality of life, including its subjective dimensions [18]. It entails the ability of older adults to maintain daily independence, participate actively in their communities, and adapt to life with chronic conditions or functional limitations [8]. The relationship between quality of life and successful aging has become an increasingly important area of research, particularly in societies with a growing proportion of older adults [19]. Implementing strategies that promote biological, psychological, and social wellbeing can enhance quality of life and support successful aging [20].

Considering the pronounced differences in resource availability, social networks, and living conditions between urban and rural areas [21], there is a need for a deeper understanding of how these contexts shape the quality of life and successful aging of older adults.

### 1.3. Integrated and Community-Oriented Approaches to Supporting Subjective Quality of Life and Successful Aging

Beyond individual biopsychosocial determinants, contemporary approaches to aging increasingly emphasize the role of integrated and community-oriented models of care and support [22] in supporting functional ability, independence, and quality of life among older adults. As aging is frequently accompanied by chronic conditions, multimorbidity, and complex care needs [5,6,7,8,9], older adults often interact with multiple health and social care providers, which places them at heightened risk of fragmented and poorly coordinated care. Fragmented care is associated with unmet needs, lower patient satisfaction, inefficient use of health services, and poorer health outcomes [23], highlighting the importance of integrated care models that ensure continuity and coordination across sectors and levels of care. In response to these challenges, the World Health Organization has promoted the Integrated Care for Older People (ICOPE) approach, which focuses on maintaining and optimizing intrinsic capacity and functional ability through person-centered, coordinated health and social services, particularly within community settings [24]. Importantly, the effectiveness of integrated care models for older adults is increasingly evaluated not only through clinical or service-related indicators, but also through patient-reported outcomes, including subjective quality of life and functional performance in everyday life. Umbrella reviews and systematic evidence syntheses identify quality of life as an outcome in the assessment of integrated and community-based care interventions for older populations [22]. These considerations are particularly relevant in contexts characterized by urban–rural disparities in service availability, accessibility, and social infrastructure, including geographical remoteness and transport-related barriers [25,26]. Additional structural barriers to care, reduced access to specialized services, and more limited social resources can shape older adults’ quality of life and opportunities for successful aging. Understanding how subjective quality of life and perceptions of successful aging vary across different living environments is therefore essential for informing the development and contextual adaptation of integrated, community-oriented approaches to supporting older adults.

### 1.4. Aims and Hypoteses

The primary aims of this study were to examine urban–rural differences in subjective quality of life and self-rated successful aging among older adults and to analyze their associations with psychosocial factors. The secondary aims were to identify predictors of successful aging, including the potential moderating effects of place of residence and chronic illness. To address these aims, the following hypotheses were formulated:

**Hypothesis 1.**  *There are significant differences in subjective quality of life, measured by the Personal Well-Being Index (PWI), and self-rated successful aging (SSAS) between older adults living in urban and rural areas.*

**Hypothesis 2.**  *Higher subjective quality of life (PWI) is associated with higher levels of self-rated successful aging (SSAS).*

**Hypothesis 3.**  *Subjective quality of life (PWI), place of residence (urban vs. rural), and the presence of chronic illness predict self-rated successful aging (SSAS).*

**Hypothesis 4.**  *Place of residence (urban vs. rural) and the presence of chronic illness moderate the relationship between subjective quality of life (PWI) and self-rated successful aging (SSAS).*

## 2. Materials and Methods

### 2.1. Study Design

This quantitative cross-sectional study across urban and rural settings was conducted between January and June 2024 in the Eastern region of Croatia—Slavonia in participants’ homes. The study is part of a larger research project on successful aging, from which a previous article has been published [27]. However, this study focuses on new analyses examining urban–rural differences in self-rated successful aging (SSAS) and subjective quality of life (PWI), their associations with psychosocial factors (community involvement and regret), and predictors of successful aging, including potential moderating effects of place of residence and chronic illness.

### 2.2. Participants

The study population comprised community-dwelling older adults, defined according to the United Nations classification as individuals aged 60 years and above [28]. Eligibility criteria included: (1) chronological age ≥ 60 years, (2) ability to provide informed consent, and (3) residence in the community, excluding individuals living in institutionalized care facilities. Participants’ place of residence was classified according to administrative boundaries in line with European statistical approaches [29]. Urban and suburban areas included settlements within city administrative boundaries or administratively connected suburban settlements, while rural areas encompassed villages.

Based on these criteria, the minimum required sample size (n = 384) was calculated using an online Sample Size Calculator [30], assuming a 95% confidence level and a 5% margin of error for a population of 181,904 older adults aged 60 years and older in the Eastern region of Croatia (Slavonia). The final sample included 403 participants, exceeding the minimum required sample size.

The final sample consisted of 175 men (43.4%) and 228 women (56.6%), with a median age of 70 years (range: 60–92). No participants identified as “Other” gender.

Power analysis confirmed that the achieved sample size provided sufficient statistical power (≥0.80) for all analyses performed, including multivariate regression (observed power = 0.95) [31].

**Figure 1:**
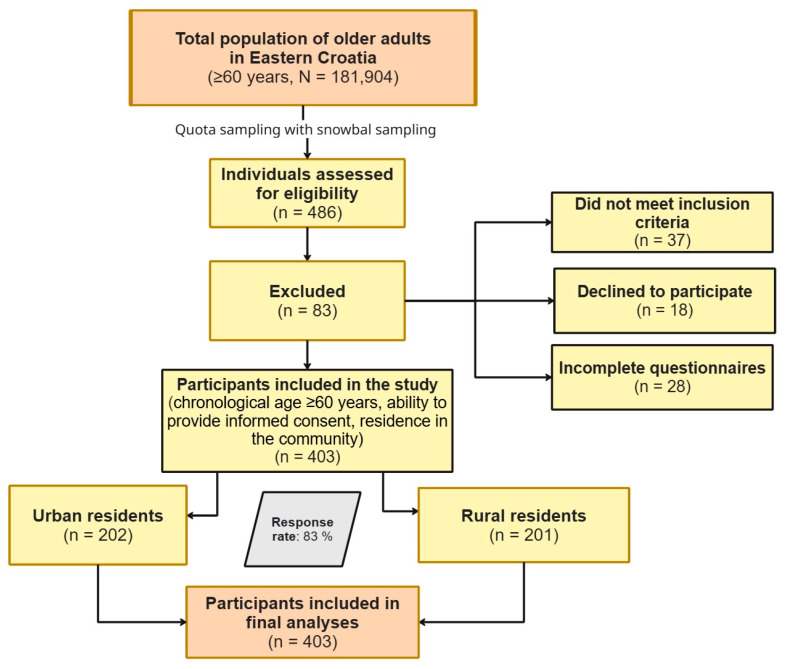
Flow diagram of participant recruitment and allocation of urban and rural older adults using quota and snowball sampling.

A non-probability quota sampling strategy was applied to ensure proportional representation of urban and rural participants, followed by snowball sampling. The response rate was 83%. The participant selection process is illustrated in Figure 1 in accordance with STROBE recommendations.

### 2.3. Instruments

The study employed validated instruments, including the Personal Wellbeing Index (PWI) [32] and the Self-assessment of Successful Aging Scale (SSAS) [33].

The PWI measures personal wellbeing through seven items of satisfaction, each corresponding to a quality of life domain: standard of living, health, achievements in life, personal relationships, sense of security, community connectedness, and future security. These domains are theoretically embedded, representing the first level of deconstruction of the global question, “How satisfied are you with your life as a whole?”. Responses are rated on an 11-point scale (0–10), ranging from “no satisfaction at all” to “completely satisfied,” with the overall score calculated as the mean of all domains. In this study, PWI scores are interpreted as reflecting the subjective dimension of quality of life, and the scale demonstrates high internal consistency (Cronbach’s α = 0.85, McDonald’s ω = 0.87).

The SSAS [34] consists of 20 items evaluating individuals’ perceptions of their own aging experience, using a five-point Likert scale (1 = “does not apply to me at all” to 5 = “fully applies to me”). The total score, calculated as the mean of all items, ranges from 20 to 100, with higher scores indicating a more positive perception of successful aging. The scale demonstrates high internal consistency (Cronbach’s α = 0.88, McDonald’s ω = 0.89).

The survey also included general and sociodemographic questions, followed by three additional items assessing participants’ perceived level of engagement in their community, regret over missed opportunities, and regret regarding past actions they now consider unwise. These items, drawn from the literature [33], relate to constructs discussed in the context of personal wellbeing and successful aging, particularly community engagement and regret as self-evaluative experiences.

### 2.4. Data Collection

Data were gathered using a non-probability quota sampling framework to achieve proportional representation of urban and rural participants, reflecting the study’s emphasis on socio-environmental determinants. Recruitment was further facilitated through a chain-referral procedure (snowball sampling technique), whereby enrolled individuals identified additional eligible participants. Data were collected in participants’ homes. Prior to fieldwork, research staff completed targeted training in communication strategies and psychological approaches relevant to engagement with older adults. Prospective participants received comprehensive written and verbal briefings on study objectives, procedures, and ethical safeguards, and provided written informed consent. Participation was fully voluntary and anonymous, with the option to withdraw at any point. Surveys were completed independently using the paper-and-pen method, with researchers providing clarification only to support comprehension without influencing responses. During the assessment of the Personal Wellbeing Index, researchers followed the authors’ administration guidelines, which require allowing respondents to skip items they do not understand and avoiding any additional explanations or examples that could shape item interpretation [32]. No time restrictions were imposed.

### 2.5. Ethical Considerations

The study was conducted in accordance with the ethical principles of the Declaration of Helsinki (1975, revised in 2013) [35]. Participation was voluntary and anonymous, and participants could withdraw at any time. Written approval was obtained from the Higher Institution Ethical Committee of the Faculty of Dental Medicine and Health Osijek (Class: 602-01/24-12/02; IRB: 2158/97-97-10-24-36). All participants received written and verbal information regarding the study’s aims, procedures, and ethical considerations, and provided written informed consent. Surveys were completed independently; researchers offered clarification only to support comprehension without guiding responses. Anonymity was maintained during and after data collection by separating the survey from the informed consent forms.

### 2.6. Data Analysis

The frequency distributions of variables were described using descriptive statistical methods. The distribution of continuous variables was assessed using the Shapiro–Wilk test, which indicated statistically significant deviations from normality (*p* < 0.05). Accordingly, measures of central tendency and dispersion were reported as medians (Me) and interquartile ranges (IQR). Differences between two independent groups were examined using the Mann–Whitney U test. Associations between variables were assessed using Spearman’s rank correlation coefficient (ρ).

Linear regression analysis using a stepwise method was conducted to examine predictors of SSAS. Prior to the regression analysis, assumptions of linearity, normality of residuals, homoscedasticity, and absence of multicollinearity were assessed and met. Although regression models were applied to examine the predictive relationships between variables, the cross-sectional nature of the study precludes causal interpretation of the observed associations. Additional moderation analyses were performed to examine whether the association between PWI and SSAS was moderated by place of residence or the presence of chronic illness. Statistical significance was set at *p* < 0.05.

All analyses were conducted using MedCalc^®^ Statistical Software version 22.023 (MedCalc Software Ltd., Ostend, Belgium), IBM SPSS Statistics version 23.0 (IBM Corp., Armonk, NY, USA), and JASP version 0.19.3 (University of Amsterdam, The Netherlands).

## 3. Results

A total of 403 participants from the eastern region of Croatia–Slavonia took part in the study. Their median age was 70 years (range 60–92), and the majority were female (n = 228, 56.6%). Participants were almost equally divided by place of residence, with 202 (50.1%) living in a city/suburban area and 201 (49.9%) living in a village. Regarding educational attainment, most participants had completed secondary education (n = 183, 45.4%), followed by primary education (n = 172, 42.7%). The majority were married (n = 210, 52.1%). Household monthly income varied, with 11 participants (2.7%) reporting no income, while the largest proportion (n = 120, 29.8%) reported income of 132.72–318.54 EUR per month. More than half of the participants (n = 206, 51.1%) reported at least one chronic illness, most commonly cardiovascular disorders (n = 108, 51.4%) and endocrine system disorders (n = 71, 34.0%), while no respondents reported integumentary or lymphatic system disorders.

Participants also rated their community involvement, regret regarding missed opportunities, and regret about past actions with a median score of 3 (IQR 2–4) for all three items (Table 1).

Between-group differences in self-perceived successful aging and quality-of-life indicators were assessed using the Mann–Whitney U test. Participants residing in rural areas reported significantly lower levels of self-assessed successful aging (median 67, IQR 58–75) compared with their urban/suburban counterparts (median 72, IQR 64–78; *p* < 0.001). They also had lower scores on the PWI (median 64, IQR 50–78 vs. 69, IQR 57–80; *p* = 0.005). Within the PWI domains, rural participants reported significantly lower satisfaction with Health (*p* = 0.02), Achieving in life (*p* = 0.01), Relationships (*p* = 0.04), Safety (*p* < 0.001), Community (*p* = 0.02), and Future security (*p* = 0.02). No significant difference was observed for the Standard of living domain (*p* = 0.06) (Table 2).

Between-group differences between male and female older adults in self-perceived successful aging and quality-of-life indicators were assessed using the Mann–Whitney U test. No statistically significant differences were observed (Table 3).

Spearman’s rank correlation coefficient (ρ) was used to assess the relationships between the scales, subscales, and participants’ age, perceived level of active involvement in the community, extent of regret for missed opportunities in life, and extent of regret for actions now considered wrong.

Age was negatively correlated with scores on the standard of living and self-rated successful aging scales (−0.145; *p* <0.001), indicating that older participants tended to report lower levels on these measures, while correlations with the PWI scale and its subscales were not significant. Perceived active involvement in the community was positively correlated with the PWI scale and all its subscales (ρ = 0.258–0.438; *p* <0.001), including standard of living, health, life achievements, close relationships, security, sense of belonging, future security, and the personal quality-of-life index. Extent of regret for missed opportunities and regret for actions now considered wrong were negatively correlated with several subscales, including life achievements, close relationships, safety, and the PWI (Table 4).

Correlation between the Self-assessment of Successful Aging Scale and the Personal Wellbeing Index (PWI) with its subscales was assessed using Spearman’s rank correlation coefficient. The Self-assessment of Successful Ageing Scale was positively correlated with the PWI total score and all subscales. Correlation coefficients ranged from moderate (ρ = 0.397–0.490) for PWI total, Achieving, Safety, and Community, to strong (ρ > 0.50) for Standard of living, Health, Relationships, and Future, with all statistically significant (*p* < 0.001) (Table 5).

Prior to conducting linear regression, assumptions of multicollinearity, normality, linearity, and homoscedasticity were assessed. Variance inflation factors (VIFs) indicated no multicollinearity among predictors (VIF < 2). Residuals were examined using Q–Q plots and scatterplots, which confirmed approximate normality and linearity. No influential cases were identified (Cook’s distance < 0.05). Subsequently, a linear regression using a stepwise method was conducted to examine predictors of SSAS. Four models were tested: the intercept-only model (M_0_), a model including only the total PWI (M_1_), a model including PWI and place of residence (0 = City/suburban, 1 = Village; M_2_), and a model including PWI, place of residence and dummy variable in presence of chronic illness (0 = No, 1 = Yes; M_3_). The intercept-only model (M_0_) is omitted as it provides no meaningful information.

M_1_ was significant and explained 45.9% of the variance in SSAS (R^2^ = 0.459, Adjusted R^2^ = 0.457; F(1, 396) = 335.51, *p* < 0.001). Higher PWI scores predicted higher SSAS scores (B = 0.608, SE = 0.033, β = 0.677, t = 18.32, *p* < 0.001).

M_2_ added place of residence and further increased explained variance to 47.8% (R^2^ = 0.478, Adjusted R^2^ = 0.475; F(2, 395) = 180.492, *p* < 0.001). Higher PWI scores remained a strong positive predictor (B = 0.589, SE = 0.033, β = 0.656, t = 17.818, *p* < 0.001), and living in a village was associated with lower SSAS scores (B = –3.232, SE = 0.856, β = –0.139, t = –3.775, *p* < 0.001).

M_3_ was significant and explained 48.3% of the variance in SSAS (R^2^ = 0.483, Adjusted R^2^ = 0.475; F(3, 394) = 122.495, *p* < 0.001). Higher PWI scores remained a strong positive predictor (B = 0.571, SE = 0.034, β = 0.636, t = 16.747, *p* < 0.001), living in a village remained associated with lower SSAS scores (B = −3.293, SE = 0.854, β = −0.142, t = −3.857, *p* < 0.001), and the presence of chronic illness was associated with lower SSAS scores (B = −1.719, SE = 0.873, t = −1.968, *p* = 0.05) (Table 6).

Additional moderation analyses were conducted to examine whether the relationship between PWI and SSAS was moderated by place of residence or the presence of chronic illness. The overall model was significant (R^2^ = 0.487, Adjusted R^2^ = 0.479, F(6, 391) = 61.820, *p* < 0.001). PWI was a significant positive predictor of SSAS (B = 0.589, β = 0.656, t = 9.145, *p* < 0.001). None of the interaction terms were statistically significant (all *p* > 0.05), and therefore, only the main effects were interpreted. Neither the presence of chronic illness (B = −1.934, t = −0.544, *p* = 0.587) nor place of residence (B = 1.235, β = 0.053, t = 0.341, *p* = 0.737) were significant predictors.

## 4. Discussion

Due to the global trend of population aging, there has been a growing emphasis on studying quality of life and successful aging among older adults [36,37]. This study specifically examined differences and factors associated with successful aging, considering the contrasts between urban and rural areas as well as accounting for the presence of chronic illness, which are theoretically expected to negatively affect successful aging [18]. In particular, the study aimed to determine whether quality of life, as a subjective dimension that can typically be enhanced through psychosocial interventions and community-based public health programs [38], represents the factor most strongly associated with successful aging, potentially independent of comorbid conditions. To address these questions, the study included equally distributed participants, with 202 (50.1%) residing in urban areas and 201 (49.9%) in rural areas. The median age was 70 years, and approximately half of the participants had chronic illness, which is expected given the increased risk of various health conditions in older age [39].

Although the conceptualization of successful aging in this study is theoretically grounded in the classical model proposed by Rowe and Kahn [18], it is important to acknowledge the limitations inherent in approaches that equate successful aging primarily with the absence of disease and high levels of functional capacity [40,41,42]. The findings of the present study partially challenge this biomedical emphasis. Subjective quality of life was independently associated with self-rated successful aging, regardless of the presence of chronic illness. This suggests that successful aging, as operationalized in this study, should not be understood solely as an outcome of optimal health status, but rather as a subjective and contextually embedded process shaped by personal wellbeing, adaptive capacities, and lived experience. These results align with contemporary shifts toward more inclusive and person-centered conceptualizations of aging well [40].

Furthermore, the observed urban–rural disparities underscore the importance of examining successful aging within broader socio-environmental contexts. The results indicate a persistent disparity between urban and rural areas, highlighting the need to examine various dimensions of quality of life and successful aging while considering sociodemographic factors that may shape these outcomes. Therefore, this discussion focuses on sociodemographic determinants, the subjective dimension of quality of life, and successful aging. Although studies often examine separate constructs, such as quality of life and personal wellbeing, their combined interpretation is justified because they are theoretically linked, with personal wellbeing considered as a subjective dimension of quality of life [32].

### 4.1. Sociodemographic Differences Between Urban and Rural Areas

The analysis of sociodemographic determinants revealed no statistically significant gender differences in personal wellbeing or in successful aging. These findings are consistent with the results reported by Vuletić and Stapić [43], who likewise observed no significant gender differences in personal wellbeing among older adults. However, evidence from other studies suggests that gender differences in quality of life [44] and successful aging [45,46] do exist. Such differences appear to be strongly shaped by cultural and social contexts, including gender roles and broader environmental conditions. Consequently, gender-related variations in self-perceptions of quality of life and successful aging are not universal but vary across countries and sociocultural settings. In the present study, chronological age was found to be significantly and negatively associated with successful aging. This finding contrasts with the results reported by Tucak Junaković et al. [47], in which age emerged as a significant positive predictor of successful aging. The authors interpreted this association by suggesting that individuals who reach advanced age may perceive longevity itself as an indicator of successful aging. The discrepancy between these findings may be partly explained by regional characteristics of the studied populations. The present study was conducted in Eastern Croatia (Slavonia), the least developed macroregion in the country [48], characterized by particularly unfavorable demographic trends compared with other Croatian regions [49]. The population of this area was heavily affected by war related losses in the early 1990s, and the age group examined in this study largely belonged to the adult population at that time. As a result, a substantial portion of their lives was spent under conditions of prolonged socioeconomic insecurity [50], which may negatively shape perceptions of one’s own aging. In contrast, the study by Tucak Junaković et al. [47] was conducted in Dalmatian counties and the City of Zagreb. According to the Organization for Economic Cooperation and Development (OECD) report [51], the City of Zagreb represents the most productive region in Croatia, while residents of Zagreb and, to a lesser extent, coastal areas tend to have higher incomes, higher educational attainment, and longer life expectancy than those living in other continental regions of Croatia. Such a context may provide a more supportive social environment for older adults, fostering greater social participation, more stable social networks, and a more positive experience of aging.

### 4.2. Urban–Rural Differences in Personal Wellbeing as the Subjective Dimension of Quality of Life

The subjective dimension of quality of life, measured using the Personal Wellbeing Index encompassing seven domains, showed notable differences between urban and rural areas. Participants residing in rural settings scored lower across most domains of subjective quality of life. These results align with the findings reported by Pekçetin et al. [52].

Consistent with the findings of lower rated health among participants from rural areas, Jiang et al. [53] report that older adults in rural areas more frequently perceive their health as poor, experience higher rates of depressive symptoms, and encounter greater difficulties in functional abilities. As a result, functional decline tends to occur earlier in rural populations than in urban ones. These rural and urban differences are largely associated with the socioeconomic status of older adults [53].

Lower satisfaction with close relationships among older adults should be interpreted in the context of urban–rural differences in social support networks. According to Iverson et al. [54], older adults in rural settings most often rely on family and friends as primary sources of support, particularly for practical daily needs, while formal services are less accessible. In contrast, participants from urban areas highlight broader access to formal and professional support resources, although some report lower satisfaction with social support and a weaker sense of safety in their neighborhoods. Social support is shaped by the specific characteristics of the environment, with older adults in rural areas relying more on informal networks and those in urban areas more on formal sources of help [54]. These differences should be considered when planning support for the older population.

In rural settings, participants reported lower levels of satisfaction with safety and future security, which may reflect limited quality and availability of services, including healthcare, as well as deteriorating environmental conditions and infrastructure [55]. Moreover, perceiving one’s immediate environment as safe is crucial for maintaining quality of life in older adulthood [56]. Feelings of insecurity among older adults are often linked to neighborhood problems and reduced trust in other residents. Those facing the most pronounced neighborhood issues report greater insecurity both in their surroundings and within their own homes, as well as an increased fear of crime [56]. Together, these findings underscore the important role of the immediate environment in shaping older adults’ subjective sense of safety.

Lower perceived life achievement among older adults reflects the combined effects of multiple interrelated factors accumulating across the life course. Older adults in rural settings often experience reduced social functioning and higher levels of social isolation, frequently linked to limited transportation options and fewer opportunities for participation in social and community activities [57]. These social constraints are accompanied by a higher burden of chronic conditions, more pronounced depressive symptoms, and greater memory difficulties, all of which are strongly associated with lower quality of life [57]. Furthermore, educational attainment in rural populations is often lower compared with urban counterparts, while higher education has consistently been associated with better quality of life and lower levels of disability in later life [58]. From a life-course perspective, these disadvantages are not isolated but accumulate over time, as conceptualized in cumulative advantage/disadvantage theory [59]. Individuals residing in rural areas may face limited educational and occupational opportunities, lower income, and restricted access to health and social services throughout their lives. Such prolonged exposure to socioeconomic adversity may contribute to lower perceived life achievement among rural older adults. Taken together, these findings suggest that lower self-rated life achievement in rural older adults is likely the product of interacting social, health, educational, and cumulative socioeconomic disadvantages.

Community belonging in rural areas is shaped by both structural constraints and social dynamics. Although rural areas are often characterized by a strong sense of mutual connectedness and greater perceived support within the local community [60], and longer-term residence contributes to stronger relationships with community members, better knowledge of the neighborhood, and a stronger sense of belonging [61], rural participants in the study reported lower levels of perceived community participation. This discrepancy can potentially be explained by structural constraints, such as greater physical isolation due to limited transportation and fewer opportunities to engage in social activities [57]. Additionally, increased migration among newer generations modifies the social composition of rural communities; the absence of neighbors during the day negatively affects older adults’ perception of belonging [60]. Economic and infrastructural limitations in rural areas further reduce opportunities for participation in organized activities [21], providing additional context for understanding the study findings. Neville et al. [62] emphasize that engagement in a rural community depends on the interconnection between physical and social environments, with transportation being a key factor for older adults’ ability to engage, a resource that is often less adapted to their needs in rural areas. Thus, differences in perceived community belonging between urban and rural settings can be interpreted within the context of interactions between resource availability and specific social relationships, both of which shape older adults’ sense of inclusion in the community.

### 4.3. Urban–Rural Differences in Successful Aging and Quality of Life

Lower levels of SSAS and wellbeing as a component of subjective quality of life among rural participants are the result of multiple factors. Song et al. [21] note that slower economic development in rural regions, combined with limited access to key resources that support an enabling environment for older adults, such as communal spaces, recreational facilities, and healthcare services, restricts their opportunities. Economic constraints in these areas reduce work and social opportunities, leading to less frequent participation in organized activities [21]. Limited access to transportation further reduces opportunities to utilize healthcare, social, and recreational services [63]. Older adults’ willingness and ability to travel to healthcare services are influenced by age, socioeconomic status, and transportation challenges, including mode of transport, costs, safety, and availability of public transit [64], which are often more pronounced in rural regions. Additionally, research suggests a higher prevalence of chronic diseases, more depressive symptoms, and memory difficulties in rural areas [57]. These findings contrast with the theoretical model of successful aging [18], which emphasizes the preservation of physical, cognitive, and social functioning along with the maintenance of high quality of life, as rural participants exhibited lower levels across all these domains. This discrepancy helps explain the lower self-rated successful aging observed among participants living in rural areas. Regression analyses further showed that PWI was strongly associated with SSAS, while rural residence and the presence of chronic illness were independently associated with lower SSAS scores. Moderation analyses showed that PWI predicts SSAS regardless of residence or chronic illness, suggesting that wellbeing consistently predicts perceived successful aging across different environmental contexts and in the presence of illness. Although rural residence and the presence of chronic illness are independently associated with lower SSAS and could theoretically attenuate the effects of personal wellbeing, the observed lack of significant moderation highlights the robustness of subjective quality of life as a context-independent determinant of perceived successful aging. Overall, these findings suggest that interventions aimed at enhancing wellbeing could help promote successful aging and warrant further investigation.

### 4.4. Psychosocial Dimensions: Community Involvement and Regret for Missed Opportunities and Past Actions

Active community involvement is positively associated with both higher quality of life and greater self-rated successful aging, emphasizing its role as a resource that allows older adults to actively influence their wellbeing and aging process despite differing life circumstances. Engagement in social and cultural activities reduces loneliness, depression, and anxiety, thereby enhancing overall functioning [65]. Conversely, regret, whether for missed opportunities or past actions, is linked to lower life satisfaction, poorer health outcomes [66,67], and reduced self-rated successful aging [68]. These findings highlight dual psychosocial pathways related to successful aging. Active community participation supports wellbeing and adaptive aging, whereas life regrets represent a psychological barrier. Interventions that foster engagement and address sources of regret may therefore promote successful aging in later life [69].

### 4.5. Perspectives for Clinical and Assistive Practice

The findings of this study have direct implications for the organization of integrated, person-centered care for older adults, particularly in rural settings. PWI was found to be strongly associated with SSAS across residence and chronic disease status. This suggests that interventions aimed at enhancing personal wellbeing and social inclusion may yield broad and transferable benefits across contexts and healthcare profiles. From a health management perspective, these findings support the integration of routine personal wellbeing screening into needs assessments for older adults within primary healthcare settings.

In rural areas, where subjective quality of life is generally lower, integrating healthcare, social services, and opportunities for community participation may better meet older adults’ needs. Personal wellbeing and self-rated successful aging can serve as patient-relevant, measurable outcomes for planning, monitoring, and evaluating integrated care models, aligning with outcome-oriented frameworks that prioritize domains most meaningful to older individuals, such as daily functioning and emotional wellbeing [70].

The high prevalence of chronic illness among older adults further underscores the need for coordinated care, as these individuals are more likely to utilize services from multiple providers across health and social care systems. Accordingly, the findings confirm the need for organizational mechanisms that reduce care fragmentation and strengthen collaboration through clinical networks.

The positive association between community involvement and personal wellbeing, alongside the negative impact of regret on quality-of-life domains, indicates that assistive practice should include structured psychosocial components, such as empowerment-oriented interventions, support for coping with losses, life-course evaluations, and the promotion of meaningful community roles. Evaluation of integrated care models would benefit from outcomes aligned with the Quadruple Aim framework, which emphasizes improving patient experience, population health, reducing costs, and supporting healthcare staff wellbeing [71].

### 4.6. Study Strengths, Limitations, and Recommendations

This study presents several strengths. It includes a sizable sample of older adults from both rural and urban areas, allowing robust comparisons across settings. The use of validated instruments to assess subjective wellbeing and self-rated successful aging enhances the reliability of the findings. Moreover, it provides novel insights into psychosocial and environmental determinants of successful aging within a region characterized by unique socio-cultural and demographic features.

Despite these strengths, several limitations should be acknowledged. The cross-sectional design prevents causal inferences, and the regional focus on Eastern Croatia—Slavonia, with its specific socio-cultural and demographic characteristics, limits generalizability. Reliance on self-report questionnaires may introduce social desirability bias.

In addition, this study employed a non-probability quota sampling strategy combined with snowball sampling, which may limit the representativeness of the sample. Although quota sampling was used to ensure an equal distribution of participants across relevant subgroups, the non-random nature of participant selection and the use of snowball sampling may have introduced selection bias and reduced sample heterogeneity. Furthermore, other potentially influential but unmeasured factors may have further affected sample composition. Although the sample was adequate for detecting main effects, it is recommended to test moderation results with a larger sample to rule out the possibility of small interaction effects, which is important when interpreting non-significant findings. These methodological limitations highlight important considerations for interpretation and highlight the need for further investigation.

Future research should adopt longitudinal designs, include probability-based sampling methods, expand to other regions, integrate quantitative and qualitative methods, and incorporate comparative samples of older adults residing in institutional settings to gain a more comprehensive understanding of successful aging.

## 5. Conclusions

This study provides evidence of significant urban–rural disparities in subjective quality of life and self-rated successful aging among older adults in Eastern Croatia–Slavonia. Older individuals residing in rural areas consistently reported lower levels of wellbeing across multiple life domains, as well as lower overall perceptions of successful aging. These findings suggest that successful aging is shaped not only by individual health status but also by broader environmental and social conditions associated with place of residence. Subjective quality of life was strongly associated with self-rated successful aging, explaining a substantial proportion of its variance. Despite lower scores observed among rural residents and participants with chronic illness, moderation analyses indicated that the association between wellbeing and successful aging remained consistent across environmental and health contexts. These findings underscore the robustness of personal wellbeing as a context-independent correlate of successful aging. This underscores the robustness of subjective wellbeing as a core component of successful aging in later life. Importantly, psychosocial factors played a meaningful role in shaping both quality of life and successful aging. Active involvement in the community was positively associated with higher wellbeing across all domains, whereas regret related to missed opportunities and past actions was linked to poorer outcomes. These findings highlight the interplay of adaptive and maladaptive psychosocial processes in later life and underscore the association between social participation, psychological resources, and self-perceived successful aging. Taken together, the results point to the need for integrated public health and social policies that go beyond medical care and address social participation, psychological wellbeing, and structural inequalities, particularly in rural settings. Interventions aimed at enhancing community engagement, reducing social isolation, and supporting adaptive coping strategies may contribute substantially to improved quality of life and more successful aging among older adults, particularly in socioeconomically disadvantaged and rural contexts.

## Figures and Tables

**Table 1:** Sociodemographic characteristics of respondents.

Characteristics of Respondents	Category	n (%)/Median (IQR)
Gender	Male	175 (43.4)
	Female	228 (56.6)
	Other	0 (0)
Place of residence	City/suburban settlement	202 (50.1)
	Village	201 (49.9)
Educational attainment	Primary school	172 (42.7)
	Secondary school	183 (45.4)
	Undergraduate/graduate degree	47 (11.7)
	Postgraduate degree (PhD)	1 (0.2)
Marital status	Married	210 (52.1)
	Widowed	127 (31.5)
	Divorced	33 (8.2)
	In a relationship	7 (1.7)
	Single	26 (6.5)
Household monthly income	No income	11 (2.7)
	66.36–132.57 EUR	23 (5.7)
	132.72–318.54 EUR	120 (29.8)
	318.68–450.98 EUR	98 (24.3)
	451.11–597.34 EUR	80 (19.9)
	more than 597.47 EUR	71 (17.6)
Chronic illness	Suffering from chronic illness	206 (51.1)
Type of illness (n = 206)	Cardiovascular system	108 (51.4)
	Respiratory system	18 (4.5)
	Nervous system	13 (6.3)
	Integumentary system	0
	Musculoskeletal system	29 (14.1)
	Endocrine system	71 (34.0)
	Gastrointestinal system	15 (7.3)
	Lymphatic system	0
	Urinary system	8 (2)
	Reproductive system	5 (1.2)
	Immune system	4 (1)
Chronological age	Years	70 (67–77)
Perceived level of active involvement in the community in which they live	1 = not at all; 5 = very much	3 (2–4)
Extent to which they regret missed opportunities in life	1 = not at all; 5 = very much	3 (2–4)
Extent to which they regret things they have done in life but now believe they should not have	1 = not at all; 5 = very much	3 (2–4)

**Table 2:** Differences in SSAS and PWI scores between urban/suburban and rural older adults.

Domain	Total	Urban/Suburban Area	Rural Area	*p* *
	Median (IQR)			
SSAS (20–100)	69 (61–76)	72 (64–78)	67 (58–75)	<0.001
PWI (0–100)	66 (53–80)	69 (57–80)	64 (50–78)	0.005
Standard (0–100)	60 (50–80)	60 (50–80)	60 (50–80)	0.06
Health (0–100)	60 (50–80)	60 (50–80)	50 (40–80)	0.02
Achieving (0–100)	70 (50–80)	70 (50–80)	60 (50–80)	0.01
Relationships (0–100)	80 (60–90)	80 (60–90)	70 (50–90)	0.04
Safety (0–100)	70 (50–90)	70 (60–90)	60 (50–80)	<0.001
Community (0–100)	70 (50–90)	70 (60–90)	65 (50–85)	0.02
Future (0–100)	70 (50–80)	70 (50–80)	60 (50–80)	0.02

* Differences between urban/suburban and rural areas were assessed using the Mann–Whitney U test.; SSAS—Self-assessment of Successful Aging Scale; PWI—Personal Wellbeing Index.

**Table 3:** Differences in SSAS and PWI scores between male and female older adults.

Domain	Male	Female	*p* *
	Median (IQR)		
SSAS (20–100)	69 (61–75)	70 (60.3–78)	0.38
PWI (0–100)	67 (54–80)	66 (53–80)	0.73
Standard (0–100)	60 (50–80)	60 (50–80)	0.25
Health (0–100)	60 (50–80)	60 (40–80)	0.47
Achieving (0–100)	70 (50–80)	70 (50–80)	0.73
Relationships (0–100)	80 (60–90)	80 (60–90)	0.64
Safety (0–100)	70 (50–80)	70 (50–90)	0.86
Community (0–100)	70 (50–90)	70 (50–90)	0.61
Future (0–100)	70 (50–80)	70 (50–80)	0.32

* Mann–Whitney U test; SSAS—Self-assessment of Successful Aging Scale; PWI—Personal Wellbeing Index.

**Table 4:** Associations between SSAS and PWI domains and age, community involvement, and regret measures.

	Age	Community Involvement	Regret: Missed Opportunities	Regret: Past Actions
	Spearman’s Correlation Coefficient ρ (*p*-Value)			
SSAS	−0.145 (<0.001)	0.431 (<0.001)	−0.090 (0.07)	−0.046 (0.36)
PWI	0.034 (0.49)	0.329 (<0.001)	−0.242 (<0.001)	−0.131 (0.01)
Standard	−0.05 (0.31)	0.373 (<0.001)	−0.111 (0.03)	−0.068 (0.18)
Health	−0.081 (0.10)	0.393 (<0.001)	−0.210 (<0.001)	−0.150 (<0.001)
Achieving	−0.014 (0.77)	0.258 (<0.001)	−0.132 (0.01)	−0.151 (<0.001)
Relationships	−0.054 (0.28)	0.329 (<0.001)	−0.150 (<0.001)	−0.107 (0.03)
Safety	−0.036 (0.48)	0.422 (<0.001)	−0.139 (0.01)	−0.097 (0.05)
Community	−0.05 (0.32)	0.438 (<0.001)	−0.172 (<0.001)	−0.085 (0.09)
Future	−0.051 (0.31)	0.465 (<0.001)	−0.205 (<0.001)	−0.148 (<0.001)

SSAS—Self-Assessment of Successful Aging Scale; PWI—Personal Wellbeing Index.

**Table 5:** Correlations between SSAS and PWI domains.

	The Self-Assessment of Successful Aging Scale
	Spearman’s Correlation Coefficient ρ (*p*-Value)
PWI	0.397 (<0.001)
Standard	0.560 (<0.001)
Health	0.583 (<0.001)
Achieving	0.467 (<0.001)
Relationships	0.510 (<0.001)
Safety	0.479 (<0.001)
Community	0.490 (<0.001)
Future	0.610 (<0.001)

**Table 6:** Linear regression of PWI, place of residence and chronic illness as a predictors of SSAS.

Model	Predictor	B	SE	β	t	*p*
M_1_	Intercept	40.601	1.589	–	25.552	<0.001
	PWI	0.608	0.033	0.677	18.317	<0.001
M_2_	Intercept	43.086	1.696	–	25.404	<0.001
	PWI	0.589	0.033	0.656	17.818	<0.001
	Residence *	−3.232	0.856	−0.139	−3.775	<0.001
M_3_	Intercept	44.801	1.901	−	23.562	<0.001
	PWI	0.571	0.034	0.636	16.747	<0.001
	Residence *	−3.293	0.854	−0.142	−3.857	<0.001
	Chronic illness **	−1.719	0.873	−	−1.968	0.05

* Residence: 0 = City/suburban, 1 = Village; ** Chronic illness: 0 = No, 1 = Yes. Standardized coefficients (β) reported only for continuous predictors; PWI—Personal Wellbeing Index; B = unstandardized regression coefficient; SE = standard error; t = *t*-test statistic

## Data Availability

The original data presented in the study are openly available on 507 FigShare at https://doi.org/10.6084/m9.figshare.30885308.

## Abbreviations

The following abbreviations are used in this manuscript:

PWI	Personal Wellbeing Index
SSAS	Self-assessment of Successful Aging Scale
IQR	Interquartile range
VIFs	Variance inflation factors
